# Interaction Between VLDL and Phosphatidylcholine Liposomes Generates New γ-LpE-like Particles

**DOI:** 10.1007/s11745-013-3861-8

**Published:** 2013-11-15

**Authors:** Agnieszka Ćwiklińska, Barbara Kortas-Stempak, Anna Gliwińska, Anastasis Pacanis, Agnieszka Kuchta, Małgorzata Wróblewska

**Affiliations:** Department of Clinical Chemistry, Medical University of Gdańsk, Dębinki 7, 80-211 Gdańsk, Poland

**Keywords:** Apolipoprotein E, Phosphatidylcholine liposomes, VLDL, Phosphatidylcholine, HDL-LpE, Gamma-LpE

## Abstract

One of the subfractions of HDL involved in reverse cholesterol transport is γ-LpE. It has been assumed that, like preβ-LpAI, it can be generated during the interaction between phosphatidylcholine liposomes and lipoproteins and can contribute to more efficient cholesterol efflux after the introduction of liposomes to plasma. However, there has been no evidence concerning what the sources of these particles in plasma might be. Here, we determined whether the interaction of phosphatidylcholine liposomes with VLDL and the subsequent conversions of particles could be a source of new γ-LpE particles. We found that the interaction between liposomes and VLDL affected its lipid and protein composition. The content of phospholipids increased (~96 %) while the content of free cholesterol and apolipoprotein E decreased in VLDL during the reaction with liposomes (~100 and ~24 %, respectively). New particles which did not contain apolipoprotein B were generated. Heterogeneous HDL-sized populations of particles were generated, containing phospholipids and apolipoprotein E as the sole apolipoprotein, with densities from 1.063 to 1.21 g/ml, either with γ-mobility on agarose gel and Stokes diameters from 8.58 to 22.07 nm or with preβ-mobility and Stokes diameters from 9.9 to 21.08 nm. The obtained results contribute to the understanding of changes in lipoproteins under the influence of phosphatidylcholine liposomes, showing the formation of new (γ-LpE)-like and (preβ-LpE)-like particles, similar in mobility and size to plasma HDL-LpE. These newly generated particles can claim a share of the antiatherogenic effects of liposomes, observed in studies both in vitro and in vivo.

## Introduction

Atherosclerosis is a major cause of death in Western countries [1]. Critical in the pathogenesis of this disease is the presence of cholesterol-laden macrophages in the arterial walls [2]. Antiatherogenic effects are produced by an uptake of the excess cholesterol from these cells and its transport to the liver in a process called reverse cholesterol transport (RCT) [3]. The largest share in RCT is assigned to apoAI-containing HDL [4]. However, it is known that HDL are a heterogeneous group of particles and subfractions which do not contain apoAI are also involved in RCT [5]. One of these subfractions is apoE-containing HDL [6].

Apolipoprotein E (apoE) is an arginine-rich, 34.2 kDa glycoprotein with pleiotropic functions including promotion of the uptake of triacylglycerol-rich lipoproteins from the circulation, maintaining macrophage lipid homeostasis, activating enzymes involved in lipoprotein metabolism and stimulating RCT (reviewed in [7]). It belongs to the group of exchangeable apolipoproteins which can move between different classes of lipoproteins in plasma [8]. Most plasma apoE is a surface component of lipoproteins containing apoB or apoA (chylomicrons, VLDL and HDL), but there is also a class of lipoproteins called HDL-LpE, in which apoE is the sole apolipoprotein. These lipoproteins measure from 9 to 18.5 nm and have densities above 1.21 g/ml. They show different mobility on agarose gel and thus γ-LpE, preβ-LpE and α-LpE have been distinguished [9]. It has been found that γ-LpE is very effective in the uptake of cholesterol from cells. After incubation of human plasma with fibroblasts loaded with [^3^H]cholesterol, the radioactivity found in γ-LpE was higher than that found in lipid-poor apoAI-containing HDL (preβ-LpAI) [10–14]. γ-LpE is a heterogenous group of particles rich in sphingomyelin [15]. It appears in plasma in very low concentrations and its metabolism is very fast; thus, its generation and metabolism have not been fully elucidated [15, 16]. Krimbou et al. [15] found that the lipolysis of VLDL may be the source of γ-LpE and it is possible that a role in maintaining the γ-LpE level in plasma is played by cell membrane phospholipids.

A number of in vivo and in vitro experiments have proved that the effectiveness of RCT can be enhanced by phosphatidylcholine liposomes [17–22]. However, the mechanisms of the changes occurring in the plasma after the introduction of the liposomes are not fully understood [23, 24]. Most attention has been paid to the interaction between liposomes and HDL resulting in changes in HDL composition and, inter alia, the generation of new preβ-LpAI particles [17, 20, 21, 25, 26]. However, there have been several reports demonstrating that lipoproteins other than HDL and apolipoproteins other than apoAI are also involved in the interaction between lipoproteins and liposomes and it is possible that the products of this interaction contribute to the antiatherogenic properties of phosphatidylcholine liposomes [17, 27–30].

The aim of this study was to determine whether the interaction between VLDL and phosphatidylcholine liposomes could be a source of new γ-LpE particles. This goal was achieved through the incubation of human VLDL with egg yolk phosphatidylcholine liposomes, followed by the separation of the reaction products and their identification using, inter alia, two-dimensional non-denaturing polyacrylamide gradient gel electrophoresis (2D-PAGGE). Our experiments have shown that interaction between VLDL and phosphatidylcholine liposomes resulted in changes in the composition and properties of particles taking part in the reaction and caused the generation of a heterogenous group of new HDL-sized range apoE-containing particles with γ- and preβ-mobility on agarose gel electrophoresis.

## Materials and Methods

### VLDL Isolation

Sera were obtained from blood drawn from apparently healthy donors following an overnight fast of 12–16 h. The serum lipid cutoff values were as follows: total cholesterol (TC) < 200 mg/dl, triacylglycerols (TAG) < 150 mg/dl, HDL-C > 40 mg/dl.

The VLDL fraction was isolated from pooled sera by the ultracentrifugation procedure described by McEneny et al. [31] with modifications. Briefly, 1.8 ml of sera was placed into an open-top centrifuge tube (Beckman Coulter Poland) and gently overlaid with 1.2 ml of normal saline (*d* = 1.006 g/ml). Ultracentrifugation was performed in a Beckman tabletop ultracentrifuge with a TLA-100.3 fixed-angle rotor (parameters: 90 min, 4 °C, 541,000×*g*). The top 1.2-ml portion was carefully collected and dialyzed against a 10 mM Na phosphate buffer pH 7.6 + 0.25 mM EDTA + 0.02 % sodium azide [32] (18 h, 4 °C).

### Liposome Preparation

Small unilamellar liposomes (SUV) were prepared from egg yolk phosphatidylcholine by sonication (Sonopuls HD 2070 ultrasonic homogenizer, Bandelin Electronic, Germany) as described earlier [28] with the following modification: liposomes were suspended in a 10 mM Na phosphate buffer pH 7.6 + 0.25 mM EDTA + 0.02 % sodium azide.

### Incubation of VLDL with Liposomes

Reaction mixtures (VLDL + PhL) were prepared by mixing VLDL and phosphatidylcholine liposomes (PhL) at a VLDL-phospholipid (PL) to PhL-PL 30:1 w/w ratio. Control mixtures were prepared by mixing VLDL with a respective volume of 10 mM Na phosphate buffer pH 7.6 + 0.25 mM EDTA + 0.02 % sodium azide corresponding to the volume of liposomes added to the reaction mixtures. These mixtures were incubated at 37 °C for 2 h. After incubation the mixtures were cooled by placing them on ice for 5 min, after which immunoprecipitation, ultracentrifugation or electrophoretic procedures were promptly performed.

### Immunoprecipitation

Immunoprecipitation was performed according to the procedure described by Nielsen et al. [33] with modifications. Briefly, 175 μl of polyclonal rabbit anti-human apoB antibody (DAKO, Poland) was added to 350-μl reaction and control mixtures and incubated overnight at 4 °C. The precipitate containing VLDL was separated by centrifugation (40 min, 4 °C, 5,000×*g*) and the supernatant was gently aspirated. The precipitate was washed with ice-cold phosphate-buffered saline and resuspended in 525 μl of 0.01 M Tris–HCl buffer pH 7.4 + 1 M NaCl. The mean recovery of lipid constituents determined in supernatants and precipitates after immunoprecipitation was 90 %.

### Ultracentrifugation

In each of 6 bell-top centrifuge tubes (Beckman Coulter Poland), 1 ml of reaction mixture was placed and overlaid with 2.2 ml of normal saline (*d* = 1.006 g/ml). Ultracentrifugation was performed in a Beckman tabletop ultracentrifuge with a TLA-100.3 fixed-angle rotor (parameters: 2 h 15 min, 4 °C, 541,000×*g*). The top fractions (*d* < 1.006 g/ml) containing VLDL (fraction* 1*) were separated by slicing. The bottom fractions were pooled and adjusted to a density of 1.063 g/ml with solid KBr, aliquoted to 6 bell-top centrifuge tubes, overlaid with KBr solution (*d* = 1.063 g/ml) and centrifuged (Beckman tabletop ultracentrifuge with a TLA-100.3 fixed-angle rotor; parameters: 3 h, 4 °C, 541,000×*g*). The top fractions, with densities below 1.063 g/ml (fraction* 2*), and the bottom fractions, with densities above 1.063 g/ml (fraction* 3*), were separated by slicing. Fractions* 1*,* 2* and* 3* were dialyzed against a 10 mM Na phosphate buffer pH 7.6 + 0.25 mM EDTA + 0.02 % sodium azide (18 h, 4 °C) and fractions* 2* and* 3* were concentrated with Stirred Ultrafiltration Cells (Amicon INC, USA). The mean recovery of lipid and protein constituents determined in fractions after ultracentrifugation was 74 %. The percentages of distribution of PL, TC and apoE content in fractions obtained by ultracentrifugation were similar to the distribution obtained after immunoprecipitation (Table 1).

**Table 1:** Comparison of the distribution of content of components (%) after separation of the reaction products by ultracentrifugation and immunoprecipitation (*n* = 7)

	Separation method	Percentage (%) (mean ± SD)		
		PL	TC	apoE
VLDL	Ultracentrifugation (fraction* 1*)	5.8 ± 1.0	52.1 ± 5.9	74.4 ± 6.8
	Immunoprecipitation (precipitate)	5.9 ± 0.6	54.1 ± 3.8	76.5 ± 7.2
Reaction products not containing apoB	Ultracentrifugation (fraction* 2* + fraction* 3*)	94.2 ± 1.0	47.9 ± 5.9	25.6 ± 6.8
	Immunoprecipitation (supernatant)	94.1 ± 0.6	45.9 ± 3.8	23.5 ± 7.2

*PL* phospholipids, *TC* total cholesterol, *apoE* apolipoprotein E

### Agarose Electrophoresis

Samples (5–20 μl) were separated by electrophoresis in agarose gel (0.75 % w/v, 100 mM Tris–barbital buffer pH 9.4, 10 °C, 130 V, 90 min). Lipoproteins were visualized by staining with Sudan Black B or transferred to a PVDF membrane (Immobilon-P Transfer Membrane, Merck Millipore Poland) by passive transfer. Lipoproteins containing apoE or apoB were detected with rabbit polyclonal antibodies to human apoE or apoB (DAKO, Poland) and anti-IgG conjugated with alkaline phosphatase (Sigma-Aldrich, Poland) using NBT/BCIP as chromogenic substrates.

### Two-Dimensional Non-Denaturing Polyacrylamide Gradient Gel Electrophoresis (2D-PAGGE)

Lipoproteins in the HDL range in reaction mixtures and fraction* 3* were separated by two-dimensional non-denaturing polyacrylamide gradient gel electrophoresis (2D-PAGGE). Briefly, 100–120 μl of reaction mixtures or fraction* 3* were separated in the first dimension by agarose electrophoresis (parameters as described above) and in the second dimension by non-denaturing polyacrylamide gradient gel electrophoresis (2–25 %, 10 °C, 160 V, 16 h). A High Molecular Weight Native Marker Kit (GE Healthcare, UK) was run as a standard on each gel. Lipoproteins separated in the gel were electrotransferred (4 °C, 30 V, 26 h) onto a PVDF membrane and apoE-containing lipoproteins were detected as described above.

### Denaturing Gradient Gel Electrophoresis (SDS-PAGE)

Samples were mixed with 10 % Triton X-100 (1:1 v/v), vortexed and incubated for 1 h at 56 °C. Then, 10–23 μl of solubilized samples were mixed with SDS-containing incubation buffer with or without a reducing agent (β-mercaptoethanol). In the case of reducing conditions application, samples were boiled with SDS-incubation buffer containing reducing agent for 5 min at 95 °C. Next, samples were separated in the SDS-polyacrylamide gradient gel (10–20 %, 155 V, 90 min). Low Molecular Weight Protein Standard (Bio-Rad Poland) was run on each gel. Proteins were visualized by staining with silver or electrotransferred to a PVDF membrane (30 V, 4 °C, 8 h) and apoE was detected as described above.

### Lipid Analysis

Concentration of lipids were measured using commercially available enzymatic kits: phospholipids (PL) purchased from Wako Diagnostics (USA); total cholesterol (TC) and triacylglycerols (TAG) purchased from Pointe Scientific (Poland); free cholesterol (FC) purchased from DiaSys (Germany). Concentration of cholesteryl esters (CE) was calculated from the difference between TC and FC.

### Apolipoprotein Analysis

The concentrations of apoE and apoB in the reaction mixtures and fractions* 1*,* 2* and* 3* were measured by immunonephelometry (Siemens Healthcare Diagnostics, Germany). The concentration of apoE in supernatants after immunoprecipitation was measured by immunoelectrophoresis (Sebia, France). Samples rich in PL were solubilized prior to analysis with Triton X-100 as described above.

### Statistical Analysis

Statistical analyses were performed using GraphPad Prism 4.03 (GraphPad Software Inc. USA). The Shapiro–Wilk test for normality was used. Statistical significance was determined by paired *t* test. Values of *p* < 0.05 were considered statistically significant.

## Results

### Changes in VLDL After Incubation with Phosphatidylcholine Liposomes

**Fig. 1:**
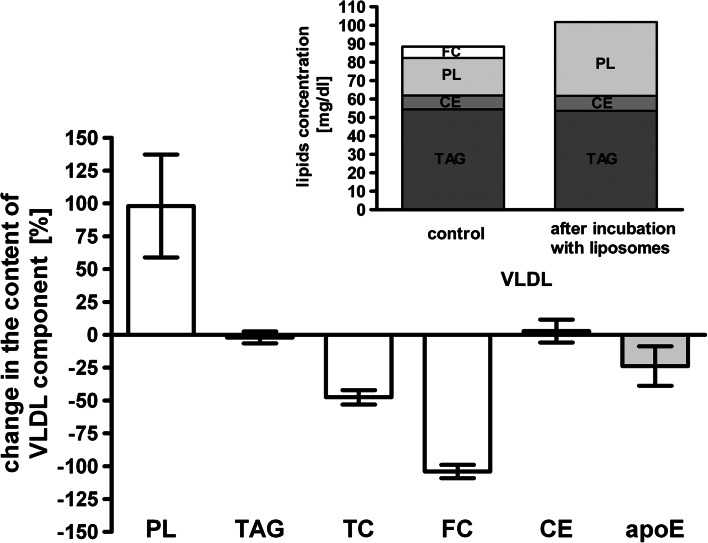
The changes of lipids and apoE content in VLDL after incubation with phosphatidylcholine liposomes. *Insert* The average concentration of the lipid components in VLDL after incubation with phosphatidylcholine liposomes. VLDL was incubated with phosphatidylcholine liposomes (VLDL-PL to PhL-PL ratio 1:30, 2 h, 37 °C). Controls were prepared by mixing VLDL with an appropriate aliquot of buffer corresponding to the volume of liposomes added to the reaction mixture (VLDL + PhL). After incubation VLDL was immunoprecipitated as described in “Materials and Methods” and supernatants and precipitates were analysed for lipids and apoE content. Changes in lipids and apoE content are expressed in relation to the content of the constituent in the control mixture. Data are expressed as means ± SD, *n* = 15

Changes in VLDL composition after incubation with liposomes was assessed after VLDL immunoprecipitation with anti-apoB antibodies and determination of lipid and apoE contents in supernatants and precipitates as described in “Materials and Methods”. The reaction between VLDL and liposomes resulted in significant changes in VLDL composition (Fig. 1). In relation to the initial content, PL in VLDL increased on average by 96 %, while FC and apoE content decreased on average by 100 and 24 %, respectively. There were no changes in TAG and CE in VLDL after the incubation. After incubation with liposomes, the content of lipids in VLDL increased on average by 15 % in comparison to their initial content (*p* < 0.0001) (Fig. 1, insert). Changes were observed only for surface lipids (PL and FC); the percentage of the surface lipid content in VLDL increased from 30 to 40 %.

For different pools of VLDL there was a wide variation in the amounts of accepted PL (CV ± 40 %) and lost apoE (CV ± 50 %); nevertheless a significant correlation was observed between these values (*r* = 0.607; *p* < 0.005; *n* = 20).

**Fig. 2:**
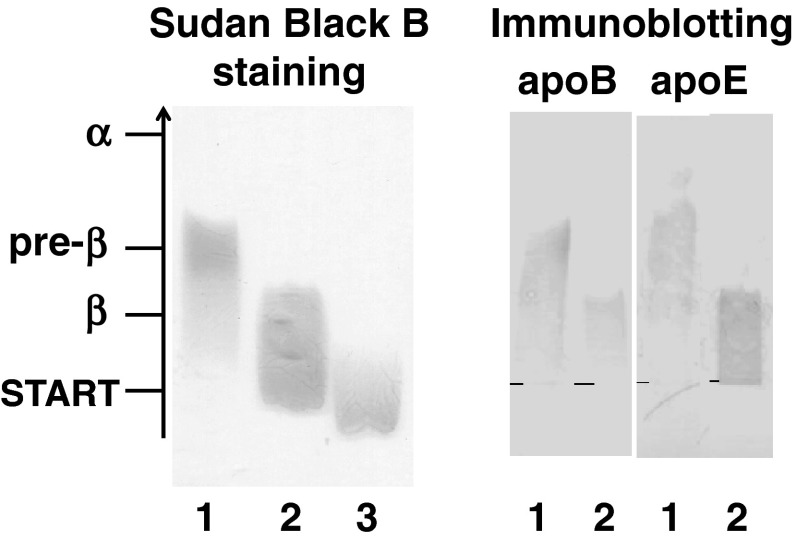
Lipoprotein fractions in the mixture of VLDL and phosphatidylcholine liposomes. VLDL was incubated with phosphatidylcholine liposomes (VLDL-PL to PhL-PL ratio 1:30, 2 h, 37 °C). Control VLDL contained an appropriate amount of VLDL but no liposomes; control liposomes contained an appropriate amount of liposomes but no VLDL. Following incubation specimens were subjected to agarose gel electrophoresis and stained with Sudan Black B or immunodetection apoE and apoB was performed as described in “Materials and Methods”. *Lane 1* control VLDL; *lane 2* reaction mixture (VLDL + PhL); *lane 3* control liposomes

Sudan Black B staining and apoE and apoB immunodetection after electrophoresis on agarose gel for control VLDL, control liposomes and reaction mixtures (VLDL + PhL) demonstrated that after incubation, VLDL displayed β-mobility, and some apoE released from VLDL shifted to the fraction with γ-mobility on electrophoresis (Fig. 2). Control liposomes remained in the site of application or moved towards the cathode. After incubation with VLDL they moved towards the anode and displayed γ-mobility on electrophoresis (Fig. 2).

### Separation of the Reaction Products by Ultracentrifugation

**Fig. 3:**
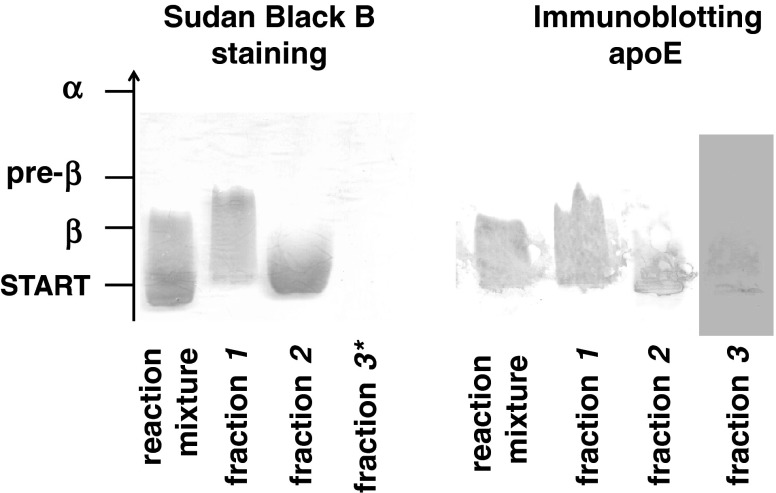
Lipoproteins in fractions obtained from the reaction mixture (VLDL + PhL) by sequential ultracentrifugation. VLDL was incubated with phosphatidylcholine liposomes (VLDL-PL to PhL-PL ratio 1:30, 2 h, 37 °C). After incubation the reaction products were separated according to their densities by ultracentrifugation as described in “Materials and Methods”. Following ultracentrifugation specimens were subjected to agarose gel electrophoresis and stained with Sudan Black B or immunodetection apoE was performed as described in “Materials and Methods”. Fraction* 1* VLDL; fraction* 2* liposomes; fraction* 3* newly generated particles with densities above 1.063 g/ml. *Lipid content in fraction* 3* was very low, hence not detectable after Sudan Black B staining

**Fig. 4:**
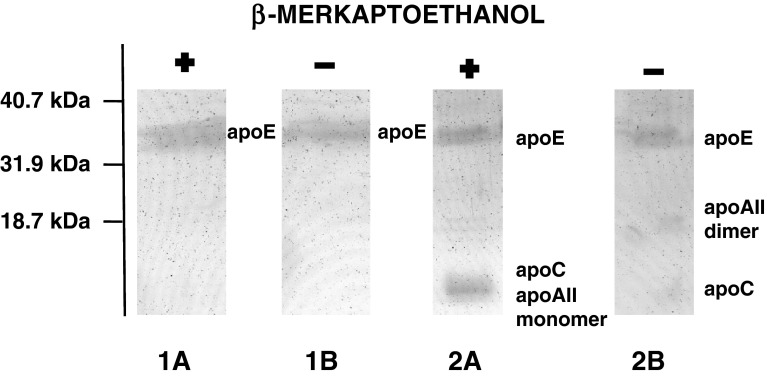
Analysis of proteins present in VLDL and fraction* 3*. VLDL was incubated with phosphatidylcholine liposomes (VLDL-PL to PhL-PL ratio 1:30, 2 h, 37 °C). After incubation the reaction products were separated according to their densities by ultracentrifugation as described in “Materials and Methods”. Next control VLDL and isolated fraction* 3* (containing newly generated particles with densities above 1.063 g/ml) were solubilized with Triton X-100, mixed with SDS-incubation buffer with or without a reducing agent (β-mercaptoethanol) and separated by SDS-polyacrylamide gradient gel (10–20 %) as described in “Materials and Methods”. Proteins were visualized with silver staining. *Lane 1* fraction* 3*, *lane 2* control VLDL. *A* Under reducing conditions, *B* no reducing conditions. *Numbers on the left* refer to the molecular mass of protein standards

**Fig. 5:**
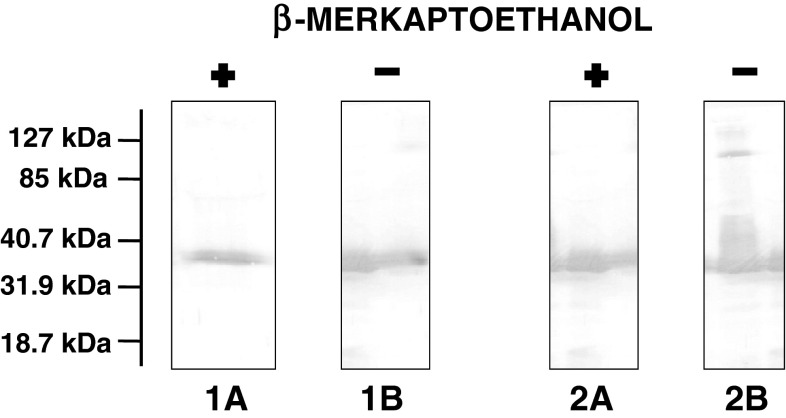
Analysis of apoE forms present in VLDL and fraction* 3*. VLDL was incubated with phosphatidylcholine liposomes (VLDL-PL to PhL-PL ratio 1:30, 2 h, 37 °C). After incubation the reaction products were separated according to their densities by ultracentrifugation as described in “Materials and Methods”. Next control VLDL and isolated fraction* 3* (containing newly generated particles with densities above 1.063 g/ml) were solubilized with Triton X-100, mixed with SDS-incubation buffer with or without a reducing agent (β-mercaptoethanol) and separated by SDS-polyacrylamide gradient gel (10–20 %). ApoE was detected with polyclonal rabbit anti-human apoE antibody as described in “Materials and Methods”. *Lane 1* fraction* 3*, *lane 2* control VLDL. *A* under reducing conditions, *B* no reducing conditions. *Numbers on the left* refer to the molecular mass of protein standards

Sequential ultracentrifugation enabled the separation of reaction products according to their densities as described in “Materials and Methods”. Three fractions were obtained: fraction* 1* with a density below 1.006 g/ml, fraction* 2* with a density from 1.006 to 1.063 g/ml and fraction* 3* with a density above 1.063 g/ml. In fraction* 1* VLDL was present, as confirmed by the presence of apoB (Table 2). It contained PL, TAG and CE as lipid components and had β-mobility on electrophoresis (Fig. 3). In fraction* 2* liposomes were present. They had γ-mobility on electrophoresis (Fig. 3) and contained PL as well as FC and apoE obtained from VLDL (Table 2). In the HDL density region (fraction* 3*) were particles containing PL (mean concentration: 0.97 ± 0.61 mg/dl) and apoE (0.20 ± 0.056 mg/l) (*n* = 7). The lipid content in this fraction was very low, hence not detectable after Sudan Black B staining. In newly-generated particles, apoE was the sole apolipoprotein obtained from VLDL, which was confirmed using SDS-PAGE (Fig. 4). ApoE in newly generated particles was in the monomeric form (mean 38.7 ± 1.1 kDa); in VLDL apoE monomer (36.3 ± 3.4 kDa) and homodimer (100.2 ± 14 kDa) (*n* = 3) were present (Fig. 5). In order to evaluate whether the density of the newly generated fraction was below or above 1.21 g/ml, fraction* 3* was adjusted to a density of 1.21 g/ml with solid KBr, overlaid with KBr (*d* = 1.21 g/ml) and centrifuged (Beckman tabletop ultracentrifuge with a TLA-100.3 fixed-angle rotor; parameters: 3 h, 4 °C, 541,000×*g*). Fractions with densities below and above 1.21 g/ml were separated by slicing and concentrated with Amicon, and PL and apoE were determined. 100 % of PL and apoE content were in fractions with densities between 1.063 and 1.21 g/ml (*n* = 3).

**Table 2:** Distribution of lipids, apoE and apoB content (%) in fractions* 1*,* 2*,* 3* obtained after separation of the reaction mixture (VLDL + PhL) by sequential ultracentrifugation (*n* = 7)

Fraction:	*1*	*2*	*3*
Density (g/ml):	<1.006	1.006 < *d* < 1.063	>1.063
Particles:	VLDL	Liposomes	Newly generated lipoproteins
Percentage (%) of the component content			
PL	5.8	94.0	0.2
TAG	100	0	0
FC	0	100	0
CE	100	0	0
apoE	73.9	24.6	1.5
apoB	100	0	0

*PL* phospholipids, *TAG* triacylglycerols, *FC* free cholesterol, *CE* cholesteryl esters, *apoE* apolipoprotein E, *apoB* apolipoprotein B

### Separation of Newly Generated Particles Using 2D-PAGGE

**Fig. 6:**
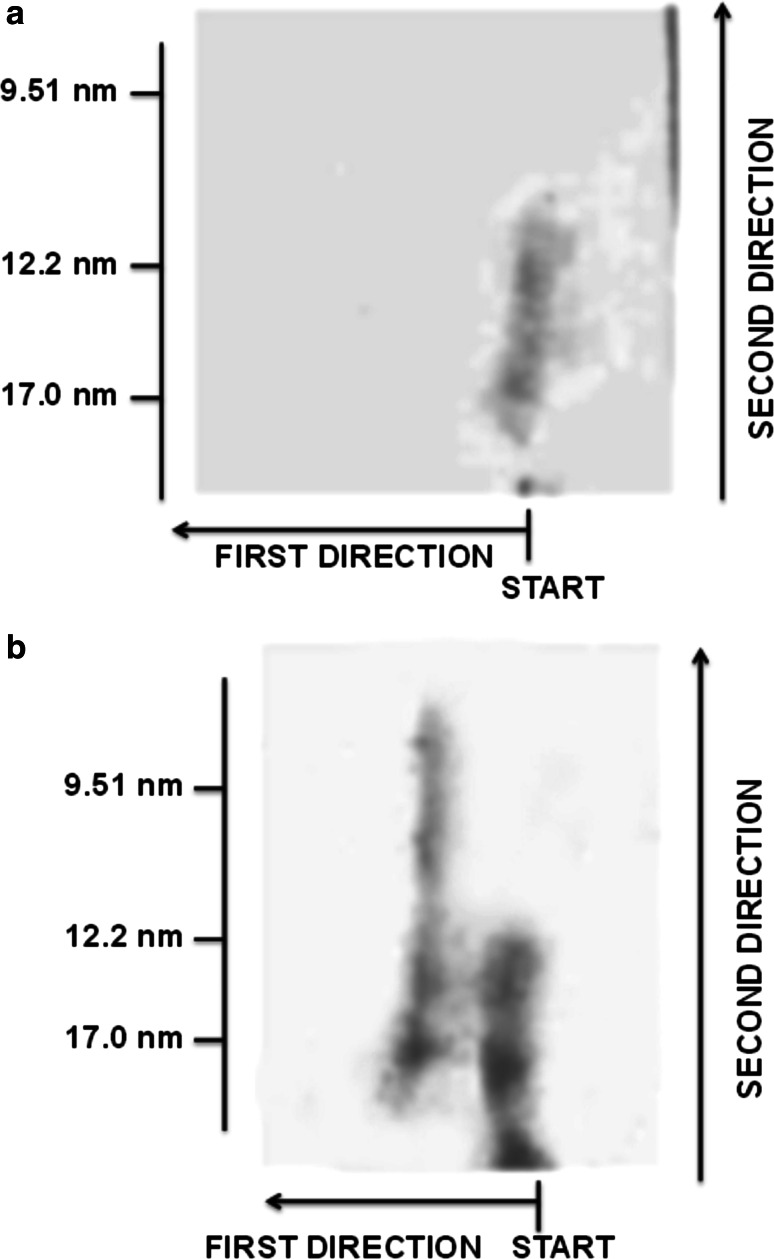
Separation of HDL-sized apoE-containing lipoproteins from reaction mixtures (VLDL + PhL) by two-dimensional non-denaturing polyacrylamide gradient gel electrophoresis (2D-PAGGE). **a** generation of particles with γ-mobility; **b** Generation of particles with γ- and preβ-mobility on electrophoresis. After incubation of VLDL with phosphatidylcholine liposomes (VLDL-PL to PhL-PL ratio 1:30, 2 h, 37 °C) aliquots of 100–120 μl were separated in the first dimension according to charge (from *right* to *left*) by agarose gel electrophoresis and in the second dimension according to particle size (from *bottom* to *top*) by non-denaturing polyacrylamide gradient gel electrophoresis (2–25 %). ApoE was detected with polyclonal rabbit anti-human apoE antibody as described in “Materials and Methods”. *Numbers on the left* refer to Stokes diameters of high molecular native marker kit proteins

Determination of the mobility and size of HDL-sized apoE-containing particles was performed using two-dimensional non-denaturing polyacrylamide gradient gel electrophoresis (2D-PAGGE) with subsequent electrotransfer and apoE immunodetection as described in “Materials and Methods”. Particles present in control VLDL, control liposomes and isolated by ultracentrifugation fraction* 1* (VLDL) and fraction* 2* (liposomes) did not migrate in 2–25 % polyacrylamide gel. Electropherograms of VLDL + PhL mixtures and isolated fraction* 3* (particles with densities above 1.063 g/ml) showed the presence of apoE-containing lipoproteins in the HDL size range with γ- or with γ- and preβ-mobility (Fig. 6).

The type and quantity of newly generated particles depended on the VLDL pool. In 4 of the 17 experiments, there was no detectable apoE-containing fraction. In 8 experiments only γ-mobility particles were observed. In the others, γ- and preβ-mobility lipoproteins were distinguished. In 4 experiments only a uniformly sized subfraction of particles was distinguished. In the others, different-sized subfractions were present. In 4 experiments, 2 different-sized populations of particles were observed; in 2 experiments, 3 populations; and in the remaining 3 experiments, 4, 5 and 6 populations.

We observed the generation of apoE-containing particles with γ-mobility and diameters from 8.58 to 22.07 nm and with preβ-mobility and diameters from 9.9 to 21.08 nm (Table 3). Since the newly generated particles were heterogenous in size, we distinguished 3 groups of particles: large particles with diameters above 16 nm (corresponding to the diameter of plasma γ_1_-LpE observed by Krimbou [15]); medium-sized particles with diameters from 11.5 to 16 nm (corresponding to γ_2_ and γ_3_-LpE); and small particles with diameters below 11.5 nm (corresponding to γ_4_ and γ_5_-LpE). Most frequently medium-sized particles with a median diameter of 13.66 nm and γ-mobility were generated. Small preβ-mobility particles with diameters of 9.9 nm were observed only in 1 experiment (Table 3).

**Table 3:** Characteristics of newly generated HDL-sized apoE-containing lipoproteins, separated from the reaction mixtures (VLDL + PhL) by two-dimensional non-denaturing polyacrylamide gradient gel electrophoresis (2D-PAGGE), *n* = 17

Electrophoretic mobility	Particles	Number of particles’ populations in 17 experiments	Stokes diameter (nm)	
			Median particle diameter	Minimum and maximum of particle diameter
γ	Large	7	18.20	16.75–22.07
	Medium	11	13.66	11.73–14.34
	Small	6	9.25	8.58–11.13
preβ	Large	3	21.08	19.15–21.08
	Medium	5	14.73	14.20–15.24
	Small	1	9.90	–

After incubation VLDL with phosphatidylcholine liposomes (VLDL-PL to PhL-PL ratio 1:30, 2 h, 37 °C) aliquots of 100–120 μl were separated in the first dimension according to charge by agarose gel electrophoresis and in the second dimension according to particle size by non-denaturing polyacrylamide gradient gel electrophoresis (2–25 %). After electrotransfer onto a PVDF membrane, apoE was detected with polyclonal rabbit anti-human apoE antibody as described in “Materials and Methods”. Electropherograms were scanned and densitometric analysis and determination of particle dimensions were performed with GelScan V. 1.45 (Krzysztof Kucharczyk Electrophoretic Techniques, Poland)

Particles isolated by 2D-PAGGE from fraction* 3* (particles with densities above 1.063 g/ml) were similar in size and mobility to particles isolated from reaction mixtures (VLDL + PhL) immediately after incubation. However, after ultracentrifugation it was more difficult to distinguish particular subfractions of particles, and a tendency towards the occurrence of larger particles was observed. In 4 experiments, in which simultaneous separation of apoE-containing particles from the VLDL + PhL and isolated fraction* 3* was performed, we distinguished in electropherograms of fractions* 3* and VLDL + PhL 12 and 15 populations of particles, respectively. The range of particle diameters for VLDL + PhL was 9.63–22.07 nm for particles with γ-mobility and 14.51–21.08 nm for particles with preβ-mobility, while for fraction* 3* it was 14.17–22.09 and 12.59–22.47 nm for particles with γ- and preβ-mobility, respectively.

## Discussion

In this study, we have found that the interaction between VLDL and phosphatidylcholine liposomes causes significant changes in VLDL and liposome composition and generates new apoE-containing lipoproteins with sizes in the HDL range. VLDL after incubation were enriched in PL and depleted in FC and apoE (Fig. 1). There were no changes in the content of VLDL core lipids TAG and CE. One effect of the interaction was an increase in total lipid content in VLDL and an increase in the percentage of surface lipids in VLDL (Fig. 1, insert). These changes, however, did not lead to the disintegration of the lipoproteins, which confirms the possibility of the isolation of VLDL after reaction by immunoprecipitation or ultracentrifugation (Table 1). Changes in composition caused changes in the electrophoretic mobility of VLDL (Figs. 2, 3). After the reaction they had β-mobility characteristic of atherogenic β-VLDL occurring in hyperlipoproteinemia type III or in animals fed a cholesterol-rich diet [34]. However, β-VLDL has less TAG and more CE relative to normal VLDL, which was not consistent with the changes in VLDL content in this study, so it is unlikely that this interaction generated the atherogenic β-VLDL, especially since after incubation of VLDL modified by liposomes with isolated HDL, VLDL lost its PL to HDL and regained preβ-mobility (data not shown). Changes observed in VLDL composition and the decrease of electrophoretic mobility of lipoproteins were similar to changes observed during the interaction between lipoproteins and liposomes by others [18, 26, 28, 30, 35]. However, other investigators have mainly indicated changes in HDL composition and their properties [17, 20, 25, 26]. It is therefore very likely that the minor changes observed during the interaction between liposomes and VLDL in the presence of all plasma lipoproteins, in comparison to changes occurring during the interaction between liposomes and isolated VLDL, did not result from the absence of this interaction in plasma but from further interactions between plasma lipoproteins, i.e. from the transfer of PL from VLDL to HDL through the action of PLTP [36].

In addition to changes in the composition and properties of VLDL, the reaction with liposomes resulted in the generation of new apolipoprotein-containing particles. Similarly Williams et al. after incubation of rabbit β-VLDL with liposomes and separation of the reaction products through hydroxyapatite chromatography, observed two distinct populations of particles containing FC and apolipoproteins other than apoB, mostly apoE [29]. Also, Guo et al. [30], after incubation of VLDL with liposomes, observed heterogenous populations of particles through an electron microscope. In this study, separation of the reaction products was performed using ultracentrifugation and two groups of particles that did not contain apoB (fraction* 2* and fraction* 3*) were distinguished (Table 1). In fraction* 2* intact liposomes were present, which had acquired FC and apoE from VLDL. Because of their size they did not enter the non-denaturing 2–25 % polyacrylamide gel. Their densities ranged from 1.006 to 1.063 g/ml and they had γ-mobility on electrophoresis (Fig. 3). Similarly Guo [30] in studies in vitro and Mendez in studies in vivo [35] reported the presence of liposomes containing FC and apoE after their interaction with lipoproteins.

The second group of particles which did not contain apoB were lipoproteins present in the fraction* 3*, with densities above 1.063 g/ml, which is characteristic for HDL (Table 2). The quantity of newly generated particles was very low, hence their presence was detectable only after at least tenfold sample concentration. They contained PL and apoE as their sole apolipoprotein (Fig. 4). ApoE was in monomeric form, while in VLDL there was monomer and homodimer apoE (Fig. 5). Also, Krimbou et al. [9] reported the presence of apoE monomer in HDL-LpE and, in his opinion, this form of exchangeable apoE may be the biologically active one. Peters-Libeu et al. [37] found that particles made of apoE and dipalmitoylphosphatidylcholine have at least two particles of monomeric apoE, while Garai et al. [38] reported that dissociation of apoE oligomers to monomers is required to bind this apolipoprotein to phospholipid vesicles.

The size of the newly generated particles enabled migration in non-denaturing 2–25 % polyacrylamide gel (Fig. 6). Particles isolated from fraction* 3* (with densities above 1.063 g/ml) by 2D-PAGGE were similar in size and mobility to particles isolated from reaction mixtures (VLDL + PhL) immediately after incubation, so their generation was not caused by using ultracentrifugation as a separation technique. However, it cannot be denied that ultracentrifugation affected particles somewhat, because it was more difficult to distinguish particular subfractions in fraction* 3*. For this reason a more reliable assessment of the size and mobility of particles was given by electropherograms obtained for the reaction mixtures (VLDL + PhL) (Table 3), because additional steps of isolation did not influence the particles’ features and VLDL and liposomes, due to their size, did not enter the gel. Neither was generation of these particles due to the isolation of VLDL by ultracentrifugation, because lipoproteins were obtained by very fast ultracentrifugation, which has little effect on lipoprotein structure and does not lead to the loss of apolipoproteins from the lipoprotein surface [39]. Furthermore Guha et al. [32] noted the stability of isolated VLDL in the phosphate buffer used in this study for as long as 7 days. Additionally, to avoid the potential influence of in vitro factors on the results, we performed incubations immediately after VLDL dialysis; all preparation procedures were performed at 4 °C and neither VLDL nor incubation mixtures were not frozen until separation and characteristics of the reaction products was completed [15]. It is also unlikely that incubation of isolated VLDL was the cause of the generation of these particles during interaction with liposomes when it is taken into account that Hajj Hassan et al. [17] reported generation of γ-LpE after incubation of liposomes with whole plasma.

During the reaction, HDL-sized apoE-containing particles with γ-mobility and diameters from 8.58 to 22.07 nm and with preβ-mobility and diameters from 9.9 to 21.08 nm were generated (Table 3). The size and mobility of these newly-formed particles corresponded to features of HDL-LpE present in human plasma [9, 15]. Similarly to research on plasma HDL-LpE [15], we observed high heterogeneity for generated populations of LpE. In 24 % of experiments we observed no new LpE particles; in 47 % we saw only particles with γ-mobility; and in the remaining 29 %, particles with γ- and preβ-mobility were present. The quantity, mobility and size of generated particles depended on the VLDL pool while the amount of exogenous phospholipids in the reaction mixture influenced only the quantity of new particles (data not shown). The type of particles generated could have been affected by the polymorphism of apoE and heterogeneity of VLDL [11, 40]. Assessment of the impact of these factors was impossible in this study, but will be performed in the next step of the research.

The following questions arise: (1) whether these newly generated LpE particles are also formed in vivo after injection of liposomes, (2) whether VLDL/liposome interaction has any physiological relevance and (3) whether newly generated LpE particles can act as acceptors of cellular cholesterol. Finding the answer to the first question may be difficult, because plasma HDL-LpE is very rapidly metabolized and particles with high PL/CE ratios are metabolized the most rapidly [16]; thus we can assume that LpE generated during interaction between VLDL and liposomes can also be rapidly metabolized in the presence of plasma components: i.e. other classes of lipoproteins, proteins and enzymes participating in lipoprotein metabolism. Indeed, we have observed the disappearance of newly generated LpE after its incubation with isolated HDL in vitro (data not shown). However, it seems very likely that VLDL/liposome interaction occurs in plasma and may have physiological relevance. Hajj Hassan et al. [17] observed generation of γ-LpE after the introduction of exogenous phospholipids to plasma. Krimbou et al. found that lipolysis of VLDL can be a source of plasma γ-LpE. He also found that the level of plasma γ-LpE was maintained when plasma was incubated with cells and he suggested that cell membrane phospholipids may play a role in γ-LpE generation in vivo [15]. The introduction of exogenous phospholipids to plasma as donors needed for the formation of new lipoproteins may therefore enhance the generation of new γ-LpE particles in vivo.

It should be also emphasized that previous studies indicated that high concentration of apoE in apoB-containing lipoproteins might be associated with hyperlipidemia and an increased risk of cardiovascular diseases [41, 42]. It was also demonstrated that lipid-lowering therapy with atorvastatin and fenofibrate leads to reduction of apoE concentration in VLDL [43, 44]. Similarly, the interaction between liposomes and VLDL caused a decrease in the apoE concentration in VLDL (Fig. 1). Furthermore, it has been shown that liposomes which acquire apoE from VLDL can block the receptor uptake of atherogenic β-VLDL by macrophages [29]. Thus it can be assumed that interaction between liposomes and VLDL and its effect on apoE distribution in lipoproteins can share in the antiatherogenic activity of liposomes.

As to the third question, it seems that newly generated LpE should be effective as an acceptors of cholesterol, since apoE enables interaction with cell receptors and activates LCAT [7]. The mechanism by which the plasma γ-LpE remove cholesterol from cells is not known. It seems that this process is not ABCA1-dependent, since lipid-free or lipid-poor apoE interacts with ABCA1 transporter [45]. It is possible that this process occurs through passive diffusion [17], but other mechanisms cannot be excluded, especially since the preferred HDL acceptors for an SR-B1-dependent cholesterol efflux pathway are large HDL particles and for an ABCG1-dependent efflux pathway are large apoE-containing HDL [5]. Moreover, the presence of apoE enhances the effectiveness of RCT, as it uniquely facilitates significant core expansion and the accumulation of large amounts of CE in lipoproteins [6]. Also, PL as a cholesterolophilic component and substrate for LCAT enhances the efficiency of cholesterol efflux from cells [22].

In conclusion, our study contributes to understanding the mechanisms of interaction between phosphatidylcholine liposomes and lipoproteins. They show that the interaction between VLDL and liposomes leads to the generation of new particles in an HDL-size range, containing apoE with γ- and preβ-mobility, which can play an important role in increasing the pool of cellular cholesterol acceptors following introduction of phosphatidylcholine liposomes to plasma.

## Abbreviations

Apo: Apolipoprotein
CE: Cholesteryl esters
FC: Free cholesterol
HDL: High density lipoprotein(s)
LpE: Lipoproteins containing apolipoprotein E
PhL: Phosphatidylcholine liposomes
PL: Phospholipids
TC: Total cholesterol
TAG: Triacylglycerol(s)
VLDL: Very low density lipoprotein(s)
